# Somatic mitochondrial DNA mutations in cancer escape purifying selection and high pathogenicity mutations lead to the oncocytic phenotype: pathogenicity analysis of reported somatic mtDNA mutations in tumors

**DOI:** 10.1186/1471-2407-12-53

**Published:** 2012-02-02

**Authors:** Luísa Pereira, Pedro Soares, Valdemar Máximo, David C Samuels

**Affiliations:** 1 Instituto de Patologia e Imunologia Molecular da Universidade do Porto (IPATIMUP), Porto, Portugal; 2 Faculdade de Medicina da Universidade do Porto, Porto, Portugal; 3 Center for Human Genetics Research, Department of Molecular Physiology and Biophysics, Vanderbilt University Medical Center, Nashville, TN, USA

## Abstract

**Background:**

The presence of somatic mitochondrial DNA (mtDNA) mutations in cancer cells has been interpreted in controversial ways, ranging from random neutral accumulation of mutations, to positive selection for high pathogenicity, or conversely to purifying selection against high pathogenicity variants as occurs at the population level.

**Methods:**

Here we evaluated the predicted pathogenicity of somatic mtDNA mutations described in cancer and compare these to the distribution of variations observed in the global human population and all possible protein variations that could occur in human mtDNA. We focus on oncocytic tumors, which are clearly associated with mitochondrial dysfunction. The protein variant pathogenicity was predicted using two computational methods, MutPred and SNPs&GO.

**Results:**

The pathogenicity score of the somatic mtDNA variants were significantly higher in oncocytic tumors compared to non-oncocytic tumors. Variations in subunits of Complex I of the electron transfer chain were significantly more common in tumors with the oncocytic phenotype, while variations in Complex V subunits were significantly more common in non-oncocytic tumors.

**Conclusions:**

Our results show that the somatic mtDNA mutations reported over all tumors are indistinguishable from a random selection from the set of all possible amino acid variations, and have therefore escaped the effects of purifying selection that act strongly at the population level. We show that the pathogenicity of somatic mtDNA mutations is a determining factor for the oncocytic phenotype. The opposite associations of the Complex I and Complex V variants with the oncocytic and non-oncocytic tumors implies that low mitochondrial membrane potential may play an important role in determining the oncocytic phenotype.

## Background

Mitochondrial DNA (mtDNA) variations have been implicated in many complex diseases, but the proof for these associations has been hard to establish [1]. One reason for the complexity is the extreme difficulty in defining a precise phenotype where the influence of mtDNA mutations can be clearly ascertained. Cancer is a good example of a complex set of diseases that have been related to mtDNA mutations [2]. Fortunately, there is a phenotype in cancer that shows a clear-cut mitochondrial involvement: the oncocytic tumor, also designated as oncocytoma, oxyphilic tumor, Hürtle cell tumor (in thyroid) and Warthin's tumor (in salivary glands). An oncocyte is a cell filled with mitochondria, and hence displaying a grainy, eosinophilic appearance and a swollen cytoplasm. This cellular phenotype can also occur in normal parathyroid glands of the elderly, in inflammatory autoimmune disorders as Hashimoto's thyroiditis, and in hyperplastic conditions as an adenomatous goiter displaying oncocytic transformation (see review in [3]). Most of these oncocytomas, which preferentially occur in the endocrine system and in some parenchymatous organs (very frequently in thyroid, kidney, salivary glands and parathyroid), are usually benign tumors displaying low invasiveness, although a few can become malignant, especially in the thyroid, where the phenotype may interfere with the intake of iodine-131 used for treatment [4,5]. Tumors can contain a mixture of cells with and without the oncocytic phenotype. The definition of a tumor as oncocytic depends on the fraction of oncocytic cells within the tumor passing a relatively high threshold. This threshold depends on the organ involved, with typical levels being 75% in thyroid, and with even stricter criteria in kidney and salivary glands, since these are generally more homogeneous neoplastic tissues [5].

Several studies have shown that oncocytic tumors accumulate a higher frequency of mtDNA mutations than non-oncocytic tumors, ranging from point substitutions, to small insertion or deletions that can lead to frameshifts or premature stop codons, and large-scale deletions, namely the common 4977 bp deletion [6-10]. The point mutations occurring in oncocytic tumors in most cases reach a homoplasmic level, and as expected since they occupy a large fraction of the mitochondrial genome, Complex I genes accumulate more mutations in oncocytic tumors compared with the other complexes having mtDNA-coded proteins (summarized in [11]), and are thought to be responsible for the impairment of oxidative phosphorylation (OXPHOS). These observations raise the question of whether these mutations contribute to the oncocytic phenotype, an issue that must be addressed in the general context of mtDNA diversity in the global population and in cancer.

Studies on the human global population have shown that mtDNA is under the effect of purifying selection, resulting in a lower proportion of non-synonymous mutations in the younger branches of the phylogenetic tree [12-15]. We have recently added quantitative information to clarify this selection [16], by using an objective measure of the depth of a node within a detailed mtDNA tree combined with a predictor of pathogenicity for non-synonymous mutations allowing one to distinguish between dangerous and almost-neutral non-synonymous mutations. That study concluded that protein variants with high pathogenicity scores are statistically significantly rarer in the older branches of the tree, a property common across the global population, represented by the macrohaplogroups L, M and N. We compared the distribution of pathogenicity scores observed on the human phylogenetic tree to the distribution of all possible protein variations to define a measure of the effect of selection on these protein variations, showing that the effect of selection increases exponentially with increasing pathogenicity score. This comparison established that the pathogenicity scoring system used, MutPred [17], could distinguish a fine gradation in pathogenicity.

The literature on the role of mitochondrial DNA mutations in cancer is in many cases contradictory. Some authors have claimed that mtDNA somatic mutations are accumulated in cancer cells due to a relaxation of the negative selection acting at the population level, thus consistent with neutrality [18]. Some mathematical models taking into account several parameters (as mtDNA point mutation fractions in a variety of human tissues) [19] showed that the homoplasy of the cancer somatic mtDNA mutations can be explained by random processes of drift, without the need to invoke positive selection for these mutations. However, other authors [20] have argued that the available data support strong selection against detrimental mtDNA mutations in tumor cells, so that intact mitochondria are required for successful tumorigenesis. Zhidkov et al. [21] analyzed two datasets of somatic cancer mutations typed by the high throughput mitochondrial sequencing array (MitoChip) concluding that the patterns of mutation in tumors are similar to the ones that occur in human evolution, so that both are shaped by similar selective constrains. These authors saw that somatic cancer mutations match the ones occurring in deep branches of the tree. Palanichamy and Zhang [22] showed that caution should be applied to the interpretation of data from MitoChip studies, as the application of phylogenetic quality control criteria led to the identification of many sample mix-ups; including in the dataset [23] which constituted 83 of the 98 samples analyzed in [21], where at least five samples had leucocytes belonging to one haplogroup and tumor to a clearly different haplogroup (sometimes as far apart as African from Eurasian haplogroups), clearly the result of sample mix-ups. Research on the role of mtDNA mutations in cancer has a long history filled with controversy; however, none of these works dealing with selection of the somatic cancer mtDNA mutations addressed the particular phenotype of oncocytic tumors, which is so clearly associated with mitochondrial dysfunction. To focus the point we consider the question of whether the predicted pathogenicity of somatic mtDNA mutations is higher in oncocytic tumors than in non-oncocytic tumors.

## Methods

### Literature search

The MedLine search was performed by using the queries "mtDNA AND cancer", "mtDNA AND oncocytic", "mtDNA AND Hurtle", "mtDNA AND oxyphilic" and "mtDNA AND Warthin". The search was performed in March 2011. For oncocytic and non-oncocytic datasets, we reviewed each publication and only used studies providing the complete mtDNA sequences, deposited in public databases, and obtained by Sanger sequencing [6,7,9]. These summed up to 101 oncocytic (16 hyperplastic thyroid nodules, 7 follicular thyroid adenomas, 22 thyroid carcinomas, 5 breast carcinomas, 9 renal oncocytomas, 25 pituitary adenomas, 16 head-and-neck tumors and 1 nasopharinx tumor) and 86 non-oncocytic (6 hyperplastic thyroid nodules, 3 follicular thyroid adenomas, 12 thyroid carcinomas, 15 breast carcinomas, 16 gliomas, 5 high-grade clear cell renal carcinomas, 20 pituitary adenomas and 9 head-and-neck tumors) samples. Data for the mtDNA variants reported in oncocytic tumors is given in Additional file 1 and data for the non-oncocytic tumors is given in Additional File 2.

For comparison to the cancers explicitly classified as oncocytic or non-oncocytic we also considered a set of mtDNA somatic mutations in general cancers, where there was no indication of a mitochondrial phenotype and for most of which there was no mtDNA sequence available for phylogenetic quality control [24-47]. For these studies we only included results from sequences obtained by Sanger sequencing, the same methodology applied in the oncocytic and non-oncocytic datasets. Data for mtDNA variants in these cancers is given in Additional file 3.

### Phylogenetic quality control

We used phylogenetic criteria [48-50] to apply a quality control to somatic mutations, as problems of sample mixing and poor quality of the material used have led to many artifacts in previous cancer mtDNA datasets [51,52]. The purpose of this focus on quality control is to assemble a dataset with both clean genotyping and phenotyping. Unfortunately, we could perform this check only in the oncocytic/non-oncocytic datasets, as full mtDNA sequence data was often missing in the other cancer studies. For this reason, the results for the other cancer class should be interpreted carefully, as some described somatic cancer mutations in this list might possibly have resulted from sample mix-up or might contain editing or sequencing errors. In the absence of the full sequences, we could not double-check this dataset for those potential errors. The phylogenetic tree used in this quality control analysis is given in Additional file 4.

Sequences from Gasparre et al. [6] were downloaded from GenBank in FASTA format and transformed in a list of polymorphisms by using the computer tool mtDNA-GeneSyn [48]. Sequences from Gasparre et al. [7] and Porcelli et al. [9] were extracted in the format of a list of polymorphisms from the public database HmtDB (http://www.hmtdb.uniba.it). We then used the online tool Haplogrep [50] to check affiliation of the samples into haplogroups. This tool is kept updated relative to the most recent haplogroup classifications based on complete mtDNA sequences and has the advantages of indicating which haplogroup-defining mutations would be expected in the sample and that could have been missed due to editing errors or real back mutations.

Some individuals in this data set miss polymorphisms that are haplogroup defining, especially in haplogroups J, T, U and I. The most problematic case is sample PA13 (PA_EU_IT_0112 in database HmtDB), which displays defining polymorphisms of haplogroups U1a1 and J, being most probably a mix-up of sequences from two individuals. Some of the few missing polymorphisms observed in those individuals can be back-mutations, but there were some only observed in the cancer tissue and not in the normal tissue, which the authors interpreted to be related with cancer; here we interpreted these last as not relevant for the tumor, being really missed mutations or back-mutations in the normal tissue. For these quality control reasons, the following mutations were discarded from the analysis: A8836G in samples HCT26 and HCT44 [6] because it is N1b haplogroup defining; T15674C in sample HCT6 [6] for being R0a2'3 haplogroup defining; A13973T in BRCA9 [6] for being T2c1a haplogroup defining; A12961G in TC6 [6] for being I5a1 haplogroup defining; G13889A in HNT10 [9] for being H4a1b haplogroup defining.

We also detected other inconsistencies between tables reported in papers and sequences deposited in the online databases. Mutation G10537A in sample HCT23 in Table 1 of Gasparre et al. [6] should be G10573A (as in GenBank Accession Number EF660990) and G4063A in sample Oncocytoma 6 in Table 1 of Gasparre et al. [7] should be G4036A (as in sample PA_XX_XX_0006 in the database HmtDB). The following mutations referred in papers are missing in the sequences deposited, but were maintained in the analyses here: T4016G in sample G5, Table 2 of Gasparre et al. [6] (EF660957 and PA_EU_IT_0045); G4831A in sample OPA11 in Porcelli et al. [9] (PA_EU_IT_0136).

### Somatic variants analyzed

In the data from the oncocytic and non-oncocytic tumors very few mutations were described in the tRNA and rRNA genes. There were three somatic variants in tRNA genes (one in *MT-TT *[10], and another in *MT-TW *and in *MT-TI *[9]) and two in rRNA genes (one in *MT-RNR2 *[10] and one in *MT-RNR1 *[7]). These small sample sizes do not allow a reliable application of statistical tests for the tRNA and rRNA genes therefore our analysis focused on the protein coding genes.

### Pathogenicity score

We aimed to predict the pathogenicity of non-synonymous mutations accumulated in oncocytic, non-oncocytic and other cancers, compared with the distribution of variations observed in the global human population and all possible protein variations that could occur in human mtDNA (through single nucleotide variations from the standard reference sequence rCRS). The MutPred score was determined in the cancer datasets as described in reference [16]. Basically, the MutPred score [17] is determined by a set of features reflecting protein structure and its dynamics, the presence of functional residues, biases of amino acid sequence, and evolutionary conservation at the substitution site and in its neighborhood. The software was trained as a random forest classification model to discriminate between disease-associated amino acid substitutions from the Human Gene Mutation Database and putatively neutral polymorphisms from Swiss-Prot.

We also used three other datasets of pathogenicity scores for nonsynonymous mtDNA variations, published previously as supplemental files in [16]. The dataset denoted as "All Possible Variants" contains pathogenicity scores for all 24,206 amino acid variants that can be generated by a single nucleotide change from the standard human mtDNA reference sequence, the rCRS [53]. The dataset denoted by "Population Variants" contained pathogenicity scores for the 2,227 nonsynonymous variants recorded in the global human mtDNA phylogenetic tree covering the L, M and N macrohaplogrops. The dataset denoted as "OMIM Pathogenic Variants" contained the pathogenicity scores for the 75 reported pathogenic mtDNA variants listed in the Online Mendelian Inheritance in Man (OMIM) database as of December 2010. This OMIM dataset was limited to reported single amino acid changes.

For comparison purposes, we estimated pathogenicity scores with another algorithm called SNPs&GO [54]. Pathogenicity scores calculated by both methods are included in Additional files: Table S1-Table S3. The pathogenicity scores from both methods were compared by nonparameteric Wilcoxon rank sum test.

## Results

The complete lists of non-silent mtDNA mutations in oncocytic, non-oncocytic and other tumors (which we refer to as "general cancer") used in this work are reported in Additional files: Table S1-Table S3 respectively. The general cancer dataset are from papers in which no mention is made of either oncocytic or non-oncocytic phenotype, so these tumors cannot be classified into either of the first two categories. The total data sum up to 67 mutations (40 of which lead to frameshifts or premature stop codons) in oncocytic tumors, 14 mutations (including 3 frameshifts or premature stop codons) in non-oncocytic tumors and 107 mutations (including 16 frameshifts or premature stop codons) in other cancers. The proportion of disruptive variations (the frameshifts and premature stop codons) is 60% in oncocytic tumors and only 21% in the non-oncocytic tumors, constituting a significant difference (*p *= 0.016) by a Fisher's exact test. This testifies to a significant accumulation of severe mutations in oncocytic tumors when compared with non-oncocytic tumors, as has been previously reported [6,7,9].

For each of the 13 mtDNA encoded protein genes we compared the number of non-silent variations (nonsynonymous, indels and premature stop codons) found in the oncocytic tumors to the number in the general cancer tumors. With only 14 non-silent variations in the non-oncocytic tumors, there was not sufficient data to break those data down by gene. For two genes there were highly significant differences in the variation frequencies in the oncocytic and general cancer categories. The occurrence of non-silent mutations in the *MT-ND1 *gene was 4.3 times higher in the oncocytic tumors than in the general cancer tumors (*p *value = 0.0006 by two-tailed Fisher's exact test). Conversely, the *MT-CO3 *gene had 0/67 non-silent variants in the oncocytic tumors but 12/107 in the general cancers (*p *value = 0.004). Even when correcting for 13 tests these two tests remain significant at a threshold *p *value of 0.05/13 = 0.004. The other eleven genes had no significant difference between the oncocytic tumors and general cancers. These statistically significant values by comparing oncocytic and general cancers strengthen the observation made previously without statistical testing [11] (where only oncocytic mutations and the ratio per gene (normalized for the gene size) were analyzed) that mutations accumulate preferentially in the *MT-ND1 *gene. The authors in that work also reported that *MT-CO1 *and *MT-ATP8 *genes seemed to be protected, a characteristic common to all other complex IV and V genes when information about the potential pathogenicity of the mutations was taken into consideration. This result is consistent with our independent observation of a significant lower mutation frequency in *MT-CO3 *gene in oncocytic tumors. Our results significantly extend these earlier observations by using a comparison of mutations reported in oncocytic tumors to mutations reported in general cancers and by showing that these differences are highly statistically significant.

The 13 proteins encoded by mtDNA are core subunits for four of the five protein complexes that make up the electron transfer chain (ETC). If we analyze the distribution of the non-silent variations by ETC complex, then there is enough data in the non-oncocytic tumors for significant results (Table 1). With 12 tests, the adjusted target significance level is 0.05/12 = 0.004. Consistent with the analysis in the previous paragraph, non-silent variants in the Complex I genes were far more likely to be found in the oncocytic tumors than in the non-oncocytic tumors. Conversely, non-silent variations were less likely in the Complex IV genes in oncocytic tumors compared to non-oncocytic tumors. There also was a significant decrease in the non-silent variants in Complex V in the oncocytic tumors compared to the non-oncocytic tumors. Only Complex III, which is represented by just a single mtDNA encoded gene, did not have a significant difference between the oncocytic and non-oncocytic tumors. The comparison of the oncocytic tumor variants with the general cancer variants gives the same pattern of significant differences, with the interesting exception of Complex V, which has no significant difference in this comparison. Finally, in the comparison between the non-oncocytic tumor variants and the general cancer variants, there was a nominally significant difference only in the Complex V genes (though this was not significant after correction for multiple testing). The picture that results from these comparisons is that non-silent mtDNA mutations in Complex I are more likely to be found in the oncocytic tumors, while non-silent variations in Complex V are more likely in the non-oncocytic tumors.

**Table 1:** Statistics for counts of non-silent mtDNA variants organized by electron transfer chain complex.

	Oncocytic vs Non-oncocytic	Oncocytic vs General Cancer	Non-oncocytic vs General Cancer
**Complex I**, **p-value**	0.003	0.00002	0.77

**Complex I**, **OR [CI]**	7.4 [2 - 27]	5.6 [2.4 - 13]	NS

**Complex III**, **p-value**	0.58	0.44	0.23

**Complex III, OR [CI]**	NS	NS	NS

**Complex IV**, **p-value**	0.034	0.0004	1

**Complex IV, OR [CI]**	0.11 [0.02 - 0.75]	0.1 [0.02 - 0.4]	NS

**Complex V**, **p-value**	0.003	0.16	0.03

**Complex V**, **OR [CI]**	0.04 [0.004 - 0.37]	NS	5 [1.3 - 19]

NS = not significant

**Figure 1:**
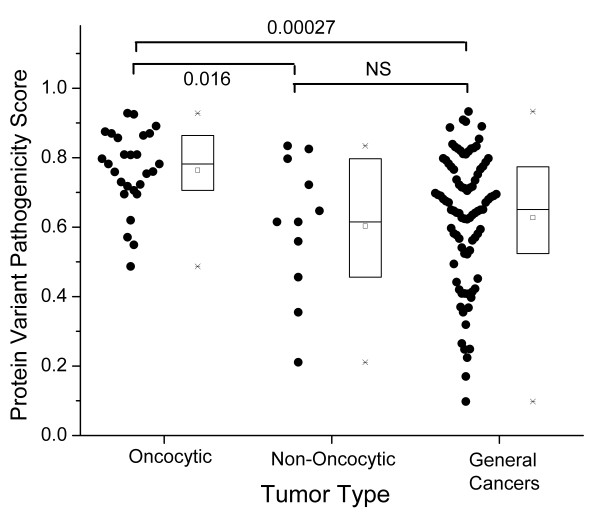
**Pathogenicity scores of mtDNA variants in oncocytic, non-oncocytic, and general cancers**. Each point represents a somatic mtDNA mutation resulting in a single amino acid change. In each category, individual data points are given to the left, and the statistics of the distribution are given in the bar chart to the right (square = mean, line = median, box = second and third quartiles, asterisk = max and min data values). P values are from nonparametric Wilcoxon rank sum tests. With three tests, the significance threshold is 0.05/3 = 0.017

While the pathogenicity of variations causing premature stop codons or frameshifts is obvious, the pathogenicity of non-synonymous variants may be highly variable, ranging from benign to highly pathogenic variations. Several methods of predicting the pathogenicity of nonsynonymous variations exist [55]. For the reported pathogenic variations resulting in a single amino acid change, we calculated predicted pathogenicity scores using the MutPred software [16,17]. The pathogenicity score in this method ranges from 0 to 1, with higher values indicating more severe pathogenicity. The nonsynonymous variations in the oncocytic tumors (Figure 1) have significantly higher median pathogenicity scores than the variations in non-oncocytic tumors (*p *= 0.016, by Wilcoxon rank sum test). The oncocytic tumors are also significantly higher in median pathogenicity score (*p *= 3 × 10^-4^) than the variations reported in general cancers. The difference between the pathogenicity scores in the non-oncocytic tumors and the general cancers is not significant, and the distribution of scores in these two categories, as shown in the box plots (Figure 1), is quite similar.

For a wider comparison we also considered three other categories of mtDNA variations; all reported pathogenic mtDNA protein variations (compiled from OMIM), all possible variations in the mtDNA encoded proteins defined by single nucleotide variations from the reference sequence rCRS [53], and all observed mtDNA-encoded protein variations reported in large human phylogenetic trees (representing the general population variants). These values were all reported in [16] and detailed explanations of their definition are given there. Briefly, we take set of OMIM variations as a set of nonsynonymous mtDNA variations with some level of proof of pathogenicity. The set of all possible variations in the mtDNA encoded proteins contains all 24206 amino acid changes that can be generated by a single nucleotide change from the rCRS. This is meant to represent the set of all possible random changes. The final group is the set of all observed non-synonymous mtDNA variants collected from human phylogenetic trees (further details of the trees are given in [16]). This group represents the population level variants in these proteins.

**Figure 2:**
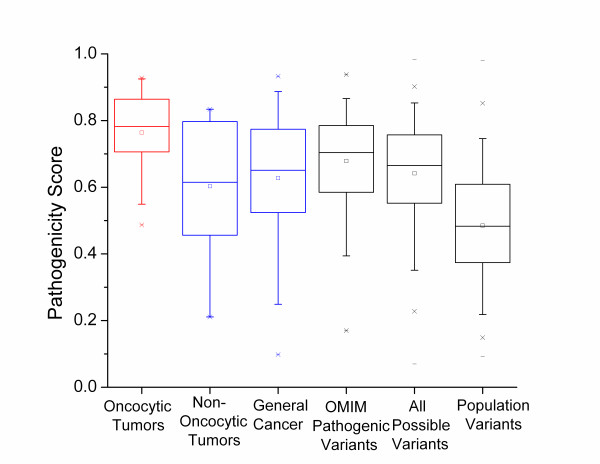
**Comparison to pathogenic mtDNA variants, all possible variants and population level variants**. The distribution of pathogenicity scores of mtDNA variations in oncocytic tumors (red) and non-oncytic and general cancer (both blue) are compared to reported mtDNA pathogenic variations, all possible variations, and normal population variations (all gray). For details on the final three categories, see the text and [16]. With five tests, the corrected significance threshold is 0.01

Figure 2 presents the distributions of the predicted pathogenicity scores for each of these categories, compared with the oncocytic and non-oncocytic tumor mtDNA variations. The median pathogenicity scores for the oncocytic tumors are significantly higher than the scores for all these categories of variations (*p *= 0.007 for oncocytic vs OMIM pathogenic variants; *p *= 1 × 10^-5 ^for oncocytic vs all possible variants; *p *= 6 × 10^-14 ^for oncocytic vs general population variants). The fact that the oncocytic tumor variants have significantly higher pathogenicity scores than the reported pathogenic mtDNA variation in OMIM emphasizes the point that the variants reported in these tumors should be considered highly pathogenic. If the somatic mtDNA variants are created randomly along the mitochondrial genome, then they should, at least approximately, be random samplings from the set of all possible variants. The fact that the oncocytic mtDNA variants have significantly higher pathogenicity scores than the set of all possible variants means that the oncocytic mtDNA variants are even worse than would be expected from random changes to the mtDNA.

The non-oncocytic tumor variants were only nominally significantly different from the general population variants (*p *= 0.026). This p-value is not low enough to survive multiple testing corrections for five tests. Conversely, the non-oncocytic tumor variations are not significantly different from the set of all possible variants (*p *= 0.6), though this lack of significance must be interpreted with care due to the small amount of non-oncocytic variant data. However it is clear that both the non-oncocytic and oncocytic mtDNA variations have for the most part escaped the purifying selection that causes the mean pathogenicity score in the population variants to be so small (Figure 2). The median pathogenicity score for the oncocytic variants is significantly higher than the median score for all possible variants while the median score for the non-oncocytic variants is smaller than that for all possible variants (though that difference does not reach significance). A reasonable interpretation of this pattern is that the somatic variations arise as a random sampling from all possible variations (at least approximately), and that those tumor cells that contain high levels of mtDNA variants with very high pathogenicity scores tend to develop the oncocytic phenotype, while those tumor cells with lower pathogenicity scores tend to maintain the non-oncocytic phenotype.

**Figure 3:**
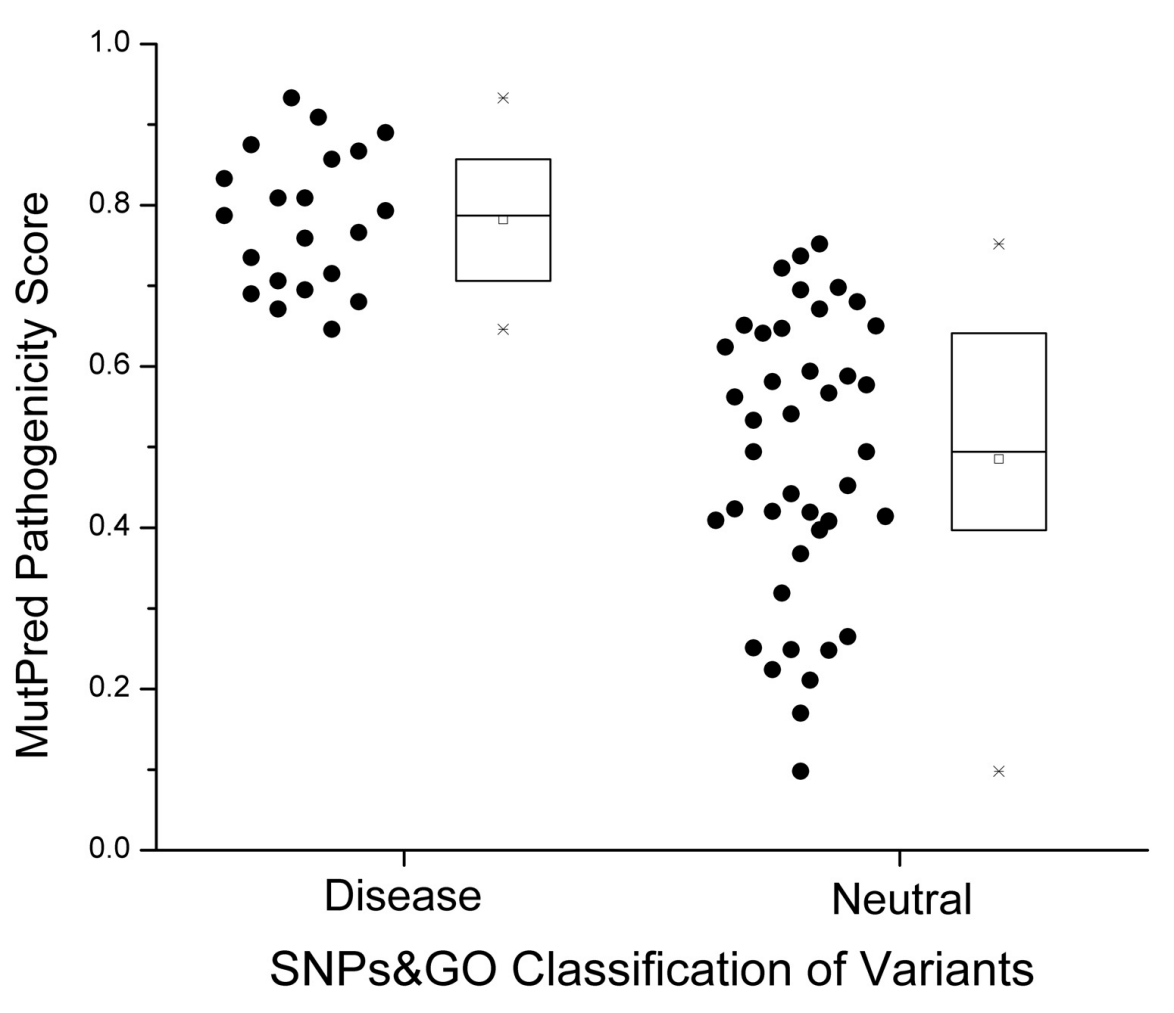
**Comparison of the pathogenicity assessment of the SNPs&GO method and the MutPred method**. All mtDNA variants in this study were assessed together. The bar plots represent the statistics of each data set as in Figure 1

MutPred is only one of many available methods for predicting the pathogenicity of nonsynonymous variants. A recent test [55] of several of these methods determined that the overall best performing methods were MutPred and SNPs&GO [54]. To test whether these results generalized to other pathogenicity scoring systems, we repeated the analysis using the SNPs&GO software. SNPs&GO classifies variants into "Neutral" or "Disease" categories, along with a reliability index ranging from 0 to 10, with high values denoting more reliable predictions. In these datasets few variants had reliability scores of 7 or higher, so we chose to only include variants with SNPs&GO reliability scores ≥ 5 in order to have a reasonably high reliability score while also having enough data to analyze. The results reported below were significant for all choices of reliability score cut-off from 0 (using all data) to 6, and there was not enough data with a reliability score above 6 to warrant testing. Our first test was to see whether the MutPred scores and SNPs&GO categories for the variants in this study were consistent. In Figure 3, we compare the MutPred pathogenicity scores for variants in the SNPs&GO "Disease" category to the MutPred scores for variants in the SNPs&GO "Neutral" category. The comparison is very highly significant (*p*-value = 4 × 10^-8 ^by nonparametric Wilcoxon rank sum test), proving that the pathogenicity assessment of these two different methods has good agreement (i.e. variants classified by SNPs&GO as "Disease" also had significantly higher MutPred pathogenicity scores on average).

Finally, we used the SNPs&GO pathogenicity analysis to compare the nonsynonymous mtDNA variations in the oncocytic, non-oncocytic and general cancer tumors. Of the 11 nonsynonymous variants in the non-oncocytic tumors, only two had SNPs&GO reliability indices ≥ 5, so there was not enough data to analyze that category using this method. In the oncocytic tumors, 7/8 nonsynonymous mtDNA variants were reliably classified by SNPs&GO as "Disease", while in the general cancer tumors only 12/46 were reliably classified as "Disease", a highly significant difference (*p*-value = 0.0018 by Fisher's exact test). Thus, the SNPs&GO analysis agrees with the MutPred analysis. Both tests conclude that mtDNA variations reported in oncocytic tumors have higher pathogenicity than the mtDNA variants reported in general cancers.

## Discussion

Oncocytic cells are not only found in cancer. They also have been reported in normal aging tissue, in inflammatory autoimmune disorders as the Hashimoto's thyroiditis, and in hyperplastic conditions as adenomatous goiter displaying oncocytic transformation (reviewed in [3]). There are currently no genetic data available for these tissues, and the only indirect evidence results from an immunohistochemistry study [56], which describes that oxyphil cells from normal parathyroid gland frequently present respiratory defects. It would be very interesting to test mtDNA variants observed in non-cancerous oncocytic cells found for instance in elderly parathyroid glands, in inflammatory autoimmune disorders and in hyperplastic conditions. This would determine if the oncocytic phenotype observed in these non-cancer tissues could also be due to highly pathogenic mtDNA mutations, as we have shown that it is in oncocytic tumor cells. Also, the comparison of the pathogenicity scores for mtDNA variants between primary and secondary oncocytic carcinomas (where the hit for hyperplasia of mitochondria occurs prior or after the hit for tumorigenesis, respectively [57]) could add valuable information to this issue. An interesting case has been described of a disruptive frameshift mtDNA mutation affecting *MT-ND5 *that was inherited at low heteroplasmy in the family of a patient where it became homoplasmic in a tumor of this individual [58]. That tumor showed an oncocytic phenotype, as we would expect from this analysis. In another study [6], the authors reported a peculiar case of a patient that presented three thyroid tumor nodules, of which only one displayed the oncocytic phenotype and the same nonsense mutation in *MT-ND5*, suggesting that this mutation could be responsible for the mitochondrial hyperplasia and hence for the Hürthle cell transformation in this case. Based on these results, we would expect that individuals carrying inherited proven pathogenic mutations, as in these cases, would be predisposed to developing the oncocytic phenotype of tumors, with the possible exception of individuals carrying pathogenic complex V protein variants who may be predisposed to the non-oncocytic tumor phenotype.

Mitochondrial hyperplasia, as occurs in the oncocytic phenotype, is generally considered to be a compensatory effect (reviewed in [11]) triggered in response to a retrograde signaling from dysfunctional mitochondria to the nucleus. The nuclear response activates the mitochondrial biogenesis pathways in order to overcome the defective OXPHOS function. In this view, mtDNA mutations have a causative role in the activation of the mitochondrial hyperplasia. Some authors [59,60] have argued that the selection of the phenotype has been driven by the micro-environment of the epithelial-cancers (where the oncocytic phenotypes are observed). Most of these cells are far away from the blood vessels in the early phases of the carcinoma, and are thus periodically under hypoxia, which will select for cells with up-regulated glucose consumption, assuming a glycolytic phenotype. These authors stress that the phenotype, not the genotype, is evolutionary selected, so that multiple mechanisms for up-regulating glucose consumption can be observed. In this case, the oncocytic phenotype could be one of several such mechanisms increasing glycolysis, but here the trigger could be environmental and not originated by the mtDNA mutations. Interestingly, when primary cultures from two thyroid tumors were established [6], each with a disruptive mtDNA mutation, both the mutations and the oncocytic phenotype were lost during culture. The authors suggested that under the culture conditions used the mtDNA mutations were under negative selection. Therefore, it seems that the *in vivo *environment of the cancer, such as hypoxia, is mandatory for the maintenance of the disruptive mutations and oncocytic phenotype.

Does this "positive selection" of the phenotype mean that those tumor cells with the high pathogenicity scores have had a better chance of survival? A commonly accepted explanation for the growth advantage in tumors relates to survival due to prevention of apoptosis, in which mitochondria play a main role. It has been shown that impairment of OXPHOS may protect cells from apoptosis [61], but it remains to be clearly shown if this happens in oncocytic tumors [4,11]. A very interesting issue is that despite the high pathogenicity of the mtDNA mutations, the impairment of OXPHOS and the oncocytic phenotype, usually (except for thyroid) indicates a tumor with low proliferative turn-over and is thus associated, in most instances, to benign neoplasms or tumors of low malignancy [5]. The idea of "adaptive landscapes" [59,60] tries to explain the acquisition of the properties of malignancy and invasion by sequential steps. Some authors have already investigated the levels of the hypoxia inducible factor-1α (HIF1α), which is activated by prolyl hydroxylases controlled by Krebs cycle metabolites (succinate and fumarate). In the oncocytic cell line XTC.UC1 a chronic destabilization of HIF1α was observed [9]. HIF1α is the main inducer of the vascular endothelial growth factor (VEGF), which regulates the generation of novel vasculature in the hypoxia environment. It seems then possible that HIF1α destabilization in oncocytic cells should occur after the homoplasmic shift of the mtDNA mutation and before neovascularization in tumor progression [9].

## Conclusions

When analyzing somatic mtDNA cancer mutations checked carefully for quality control based on phylogenetic criteria, our results showed that these variants seem to be accumulating at random from the set of all possible protein variations. This escape from the effects of purifying selection acting at the population level is most probably due to the protection of these cells from apoptosis. Based on these data it is reasonable to make the inference that the severity of the pathogenicity score of the mtDNA variants in the tumor is a major factor determining whether the tumor develops an oncocytic or a non-oncocytic phenotype. Furthermore, we confirmed that non-silent Complex I variants are found more often in the oncocytic phenotype (an observation that we now support by statistical test) while we also discovered that non-silent Complex V variants are more common in the non-oncocytic phenotype. Since the activity of Complex I raises the mitochondrial membrane potential and Complex V lowers the membrane potential, this argues for an important role of the membrane potential in the determination of the oncocytic or non-oncocytic phenotype.

## Abbreviations

mtDNA: Mitochondrial DNA; OXPHOS: Oxidative phosphorylation: HmtDB: Human mitochondrial database; rCRS: Revised Cambridge reference sequence; OMIM: Online mendelian inheritance in man; HIF1α: Hypoxia inducible factor-1α; VEGF: Vascular endothelial growth factor

## Competing interests

The authors declare that they have no competing interests.

## Authors' contributions

LP and DCS designed the work. LP and VM conducted the literature search. LP and PS applied the phylogenetic quality control. DCS performed the statistical analyses. All authors contributed to the interpretation of the results. LP and DCS drafted the manuscript, and all authors contributed to its final version. All authors have read and approved the final manuscript.

## Pre-publication history

The pre-publication history for this paper can be accessed here:

http://www.biomedcentral.com/1471-2407/12/53/prepub

## Supplementary Material

Additional file 1 **Table S1**. Table of data for the somatic mtDNA mutations reported in oncocytic tumors. Click here for file

Additional file 2 **Table S2**. Table of data for the somatic mtDNA mutations reported in non-oncocytic tumors. Click here for file

Additional file 3 **Table S3**. Table of data for somatic mutations reported in general cancer tumors. Click here for file

Additional file 4 **Table S4**. Phylogenetic tree of the cancer mtDNA sequences used for sequence QC. Click here for file
