# Supplementary material for: Somatic mitochondrial DNA mutations in cancer escape purifying selection and high pathogenicity mutations lead to the oncocytic phenotype: pathogenicity analysis of reported somatic mtDNA mutations in tumors
Source: BMC Cancer. 2012 Feb 2;12:53. doi: 10.1186/1471-2407-12-53 (PMC3342922; doi:10.1186/1471-2407-12-53)
Supplement: Additional file 1 — Table S1. Table of data for the somatic mtDNA mutations reported in oncocytic tumors. [file 1471-2407-12-53-S1.PDF]

Somatic mitochondrial DNA mutations in cancer escape purifying selection and high pathogenicity mutations lead to the oncocytic phenotype

Luísa Pereira, Pedro Soares, Valdemar Máximo and David C. Samuels

Additional Table 1 – Oncocytic tumor data. "NA" = not applicable, "X" = stop codon.

Sample	Diagnosis	Base change	AA change	Gene	MutPred Score	Reference	Haplogroup
BRCA13	invasive ductal carcinoma of the breast	3331del242bp	disruptive	*MT-ND1*	NA	Gasparre et al. (2007)	HV
BRCA14	invasive ductal carcinoma of the breast	T15843C	M366T	*MT-CYTB*	0.487	Gasparre et al. (2007)	V2
BRCA17	invasive ductal carcinoma of the breast	T15813G	V356G	*MT-CYTB*	0.549	Gasparre et al. (2007)	H1e1
HCT1	oncocytic hyperplastic thyroid module	G13414A	G360X	*MT-ND5*	NA	Gasparre et al. (2007)	H35
HCT1	oncocytic hyperplastic thyroid module	T13271C	L312P	*MT-ND5*	0.782	Gasparre et al. (2007)	H35
HCT16	oncocytic thyroid carcinoma with papillary features	3331del242bp	disruptive	*MT-ND1*	NA	Gasparre et al. (2007)	U4b1a1a
HCT18	oncocytic thyroid carcinoma with papillary features	G4975A	G169E	*MT-ND2*	0.928	Gasparre et al. (2007)	J1b2
HCT21	oncocytic hyperplastic thyroid module	3571insC	disruptive	*MT-ND1*	NA	Gasparre et al. (2007)	H13a2a
HCT23	oncocytic hyperplastic thyroid module	G10573A	G35E	*MT-ND4L*	0.695	Gasparre et al. (2007)	H14a
HCT25	oncocytic hyperplastic thyroid module	G12056A	E433K	*MT-ND4*	0.73	Gasparre et al. (2007)	T2b7
HCT26	oncocytic thyroid carcinoma	3571insC	disruptive	*MT-ND1*	NA	Gasparre et al. (2007)	N1b1d
HCT27	oncocytic thyroid carcinoma	11085-6d	disruptive	*MT-ND4*	NA	Gasparre et al. (2007)	H
HCT28	oncocytic thyroid carcinoma	G4831A	G121D	*MT-ND2*	0.809	Gasparre et al. (2007)	H
HCT28	oncocytic thyroid carcinoma	11038delA	disruptive	*MT-ND4*	NA	Gasparre et al. (2007)	H
HCT29	oncocytic thyroid carcinoma	13235insT	disruptive	*MT-ND5*	NA	Gasparre et al. (2007)	H1q
HCT30	oncocytic follicular thyroid adenoma	T15209C	Y155H	*MT-CYTB*	0.695	Gasparre et al. (2007)	H18
HCT31	oncocytic thyroid carcinoma with papillary features	G8839A	A105T	*MT-ATP6*	0.706	Gasparre et al. (2007)	H1q
HCT33	oncocytic thyroid carcinoma	G4720A	W84X	*MT-ND2*	NA	Gasparre et al. (2007)	T1a1
HCT36	oncocytic thyroid carcinoma with papillary features	G11475A	G239D	*MT-ND4*	0.87	Gasparre et al. (2007)	H5
HCT37	oncocytic thyroid carcinoma with papillary features	T3949C	Y215H	*MT-ND1*	0.76	Gasparre et al. (2007)	K1a
HCT38	oncocytic follicular thyroid adenoma	G11403A	W215X	*MT-ND4*	NA	Gasparre et al. (2007)	V1a2

HCT39	oncocytic thyroid carcinoma	G3392A	G29D	*MT-ND1*	0.864	Gasparre et al. (2007)	U2e1
HCT4	oncocytic hyperplastic thyroid module	G5185A	W239X	*MT-ND2*	NA	Gasparre et al. (2007)	T2b
HCT40	oncocytic follicular thyroid adenoma	G13042A	A236T	*MT-ND5*	0.754	Gasparre et al. (2007)	V
HCT42	oncocytic thyroid carcinoma	10885delT	disruptive	*ND4*	NA	Gasparre et al. (2007)	H2a1
HCT43	oncocytic follicular thyroid adenoma	T4222C	S306P	*MT-ND1*	0.723	Gasparre et al. (2007)	H3
HCT44	oncocytic follicular thyroid adenoma	T12797C	L154P	*MT-ND5*	0.759	Gasparre et al. (2007)	N1b1b
HCT5	oncocytic hyperplastic thyroid module	G4148A	R281H	*MT-ND1*	0.808	Gasparre et al. (2007)	H1q
HCT7	oncocytic hyperplastic thyroid module	A13870T	K512X	*MT-ND5*	NA	Gasparre et al. (2007)	HV
HCT9	oncocytic hyperplastic thyroid module	T11613C	L285P	*MT-ND4*	0.891	Gasparre et al. (2007)	HV0b
1	Benign renal oncocytoma	11872insC	disruptive	*MT-ND4*	NA	Gasparre et al. (2008)	W
2	Benign renal oncocytoma	14429delG	disruptive	*MT-ND6*	NA	Gasparre et al. (2008)	H3
3	Benign renal oncocytoma	G3664A	G120X	*MT-ND1*	NA	Gasparre et al. (2008)	L1b1a
3	Benign renal oncocytoma	C6567T	P222S	*MT-COI*	0.782	Gasparre et al. (2008)	L1b1a
5	Benign renal oncocytoma	13937delAC	disruptive	*MT-ND5*	NA	Gasparre et al. (2008)	M1b1a
6	Benign renal oncocytoma	G4036A	G244X	*MT-ND1*	NA	Gasparre et al. (2008)	U5a1
7	Benign renal oncocytoma	T13847C	L504P	*MT-ND5*	0.925	Gasparre et al. (2008)	H1
9	Benign renal oncocytoma	15342insT	disruptive	*MT-CYTB*	NA	Gasparre et al. (2008)	H4a1a1
OPA1	oncocytic pituitary adenoma	3571insC	disruptive	*MT-ND1*	NA	Porcelli et al. (2010)	H5a1
OPA2	oncocytic pituitary adenoma	11872insC	disruptive	*MT-ND4*	NA	Porcelli et al. (2010)	H1e1a
OPA3	oncocytic pituitary adenoma	G11832A	W358X	*MT-ND4*	NA	Porcelli et al. (2010)	V3
OPA4	oncocytic pituitary adenoma	12425delA	disruptive	*MT-ND5*	NA	Porcelli et al. (2010)	X2
OPA5	oncocytic pituitary adenoma	10952insC	disruptive	*MT-ND4*	NA	Porcelli et al. (2010)	H1
OPA6	oncocytic pituitary adenoma	13083insC	disruptive	*MT-ND5*	NA	Porcelli et al. (2010)	H3d
OPA6	oncocytic pituitary adenoma	14080insA	disruptive	*MT-ND5*	NA	Porcelli et al. (2010)	H3d
OPA7	oncocytic pituitary adenoma	G10386A	G110X	*MT-ND3*	NA	Porcelli et al. (2010)	T2a1b
OPA8	oncocytic pituitary adenoma	11237insC	disruptive	*MT-ND4*	NA	Porcelli et al. (2010)	K1a3a
OPA9	oncocytic pituitary adenoma	G11475A	G239D	*MT-ND4*	0.87	Porcelli et al. (2010)	HV1a
OPA10	oncocytic pituitary adenoma	3571insC	disruptive	*MT-ND1*	NA	Porcelli et al. (2010)	T2b
OPA11	oncocytic pituitary adenoma	G4831A	G121D	*MT-ND2*	0.809	Porcelli et al. (2010)	H
OPA12	oncocytic pituitary adenoma	10952insC	disruptive	*MT-ND4*	NA	Porcelli et al. (2010)	H1
OPA13	oncocytic pituitary adenoma	11872insC	disruptive	*MT-ND4*	NA	Porcelli et al. (2010)	W4a

OPA14	oncocytic pituitary adenoma	G12778A	G148X	*MT-ND5*	STOP	Porcelli et al. (2010)	U1a1
HNT10	parotid oncocytoma	5251delT	disruptive	*MT-ND2*	NA	Porcelli et al. (2010)	H4a1b
HNT11	Warthin tumor or parotid oncocytic adenoma	del232 bp	disruptive	*MT-ND5*	NA	Porcelli et al. (2010)	M1a3b
HNT12	Warthin tumor or parotid oncocytic adenoma	T3679C	S125P	*MT-ND1*	0.857	Porcelli et al. (2010)	H
HNT13	Warthin tumor or parotid oncocytic adenoma	T6210C	W103R	*MT-COI*	0.875	Porcelli et al. (2010)	T2b7
HNT15	Warthin tumor or parotid oncocytic adenoma	G3436A	G44X	*MT-ND1*	NA	Porcelli et al. (2010)	V
HNT17	Warthin tumor or parotid oncocytic adenoma	G13178A	G281D	*MT-ND5*	0.718	Porcelli et al. (2010)	H
HNT18	parotid oncocytoma	G13714A	G460X	*MT-ND5*	NA	Porcelli et al. (2010)	H
HNT19	parotid oncocytoma	G5260A	W264X	*MT-ND2*	NA	Porcelli et al. (2010)	T2b2
HNT20	Warthin tumor or parotid oncocytic adenoma	G13366A	G344X	*MT-ND5*	NA	Porcelli et al. (2010)	W1
HNT21	Warthin tumor or parotid oncocytic adenoma	T3931C	S209P	*MT-ND1*	0.62	Porcelli et al. (2010)	T2
HNT22	Warthin tumor or parotid oncocytic adenoma	C15767T	Q341X	*MT-CYTB*	NA	Porcelli et al. (2010)	V
HNT23	Warthin tumor or parotid oncocytic adenoma	T13540C	S402P	*MT-ND5*	0.571	Porcelli et al. (2010)	V
HNT24	Warthin tumor or parotid oncocytic adenoma	11621delTA	disruptive	*MT-ND4*	NA	Porcelli et al. (2010)	H
HNT25	Warthin tumor or parotid oncocytic adenoma	G3922A	E206K	*MT-ND1*	0.797	Porcelli et al. (2010)	K1a
