# Supplementary material for: Somatic mitochondrial DNA mutations in cancer escape purifying selection and high pathogenicity mutations lead to the oncocytic phenotype: pathogenicity analysis of reported somatic mtDNA mutations in tumors
Source: BMC Cancer. 2012 Feb 2;12:53. doi: 10.1186/1471-2407-12-53 (PMC3342922; doi:10.1186/1471-2407-12-53)
Supplement: Additional file 2 — Table S2. Table of data for the somatic mtDNA mutations reported in non-oncocytic tumors. [file 1471-2407-12-53-S2.PDF]

Somatic mitochondrial DNA mutations in cancer escape purifying selection and high pathogenicity mutations lead to the oncocytic phenotype

Luísa Pereira, Pedro Soares, Valdemar Máximo and David C. Samuels

Additional Table 2 – Non-oncocytic tumor data

Sample	Diagnosis	Base change	AA change	Gene	MutPred Score	Reference	Haplogroup
BRCA3	invasive ductal carcirnoma of the breast	T12601C	F89L	*MT-ND5*	0.456	Gasparre et al. (2007)	HV1a2
BRCA10	invasive lobular carcinoma of the breast	T9903C	F233L	*MT-COXIII*	0.834	Gasparre et al. (2007)	X2
BRCA5	invasive ductal carcirnoma of the breast	T9119C	L198P	*MT-ATP6*	0.722	Gasparre et al. (2007)	N1c
G5	astrocytoma	T4016G	L237R	*MT-ND1*	0.825	Gasparre et al. (2007)	U2e1
G15	astrocytoma	T11204C	F149L	*MT-ND4*	0.615	Gasparre et al. (2007)	J2a2a
TC7	hyperplastic thyroid module	T11204C	F149L	*MT-ND4*	0.615	Gasparre et al. (2007)	H13a1a1
TC12	papillary thyroid carcinoma	T11736C	L326P	*MT-ND4*	0.797	Gasparre et al. (2007)	H1
TC19	papillary thyroid carcinoma	10116delAT	disruptive	*MT-ND3*	NA	Gasparre et al. (2007)	U5a2b1
TC8	follicular thyroid carcinoma	G3842A	W179X	*MT-ND1*	NA	Gasparre et al. (2007)	H1
TC18	papillary thyroid carcinoma	C7441A	S513Y	*MT-COXI*	0.211	Gasparre et al. (2007)	J1c2c1
TC16	papillary thyroid carcinoma	A8725G	T67A	*MT-ATP6*	0.559	Gasparre et al. (2007)	K1a2
TC4	hyperplastic thyroid module	G8572A	G16S	*MT-ATP6*	0.355	Gasparre et al. (2007)	J1c
HNT5	high-grade ductal carcinoma	7406insACC	502insP	*MT-CO1*	NA	Porcelli et al. (2010)	H4a
HNT8	pleomorphic adenoma	A8803G	T93A	*MT-ATP6*	0.647	Porcelli et al. (2010)	H1c
