# Supplementary material for: Somatic mitochondrial DNA mutations in cancer escape purifying selection and high pathogenicity mutations lead to the oncocytic phenotype: pathogenicity analysis of reported somatic mtDNA mutations in tumors
Source: BMC Cancer. 2012 Feb 2;12:53. doi: 10.1186/1471-2407-12-53 (PMC3342922; doi:10.1186/1471-2407-12-53)
Supplement: Additional file 3 — Table S3. Table of data for somatic mutations reported in general cancer tumors. [file 1471-2407-12-53-S3.PDF]

Somatic mitochondrial DNA mutations in cancer escape purifying selection and high pathogenicity mutations lead to the oncocytic phenotype

Luísa Pereira, Pedro Soares, Valdemar Máximo and David C. Samuels

Additional Table 3 – Other cancer

Sample	Diagnosis	Base change	AA change	Gene	MutPredScore	Reference	Sequencing	Size of molecule surveyed
PCA013	Prostate carcinoma	T3394C	Y30H	*MT-ND1*	0.783	Kloss-Brandsatter et al. (2010)	Sanger sequencing	Complete
PCA012	Prostate carcinoma	T4522C	L18P	*MT-ND2*	0.687	Kloss-Brandsatter et al. (2010)	Sanger sequencing	Complete
PCA022	Prostate carcinoma	G5031A	G188X	*MT-ND2*	NA	Kloss-Brandsatter et al. (2010)	Sanger sequencing	Complete
PCA007	Prostate carcinoma	G6384A	A161T	*MT-CO1*	0.647	Kloss-Brandsatter et al. (2010)	Sanger sequencing	Complete
PCA015	Prostate carcinoma	G8184A	C200Y	*MT-CO2*	0.89	Kloss-Brandsatter et al. (2010)	Sanger sequencing	Complete
PCA027	Prostate carcinoma	T9116C	I197T	*MT-ATP6*	0.671	Kloss-Brandsatter et al. (2010)	Sanger sequencing	Complete
PCA038	Prostate carcinoma	G9157A	A211T	*MT-ATP6*	0.671	Kloss-Brandsatter et al. (2010)	Sanger sequencing	Complete
PCA018	Prostate carcinoma	G9438A	G78S	*MT-CO3*	0.839	Kloss-Brandsatter et al. (2010)	Sanger sequencing	Complete
PCA038	Prostate carcinoma	T9927C	Y241H	*MT-CO3*	0.775	Kloss-Brandsatter et al. (2010)	Sanger sequencing	Complete
PCA025	Prostate carcinoma	T9930C	W242R	*MT-CO3*	0.793	Kloss-Brandsatter et al. (2010)	Sanger sequencing	Complete
PCA008	Prostate carcinoma	T11139C	I127T	*MT-ND4*	0.623	Kloss-Brandsatter et al. (2010)	Sanger sequencing	Complete
PCA019	Prostate carcinoma	G11391A	G211E	*MT-ND4*	0.904	Kloss-Brandsatter et al. (2010)	Sanger sequencing	Complete
PCA001	Prostate carcinoma	T11921C	W388R	*MT-ND4*	0.887	Kloss-Brandsatter et al. (2010)	Sanger sequencing	Complete
PCA023	Prostate carcinoma	G13718A	S461N	*MT-ND5*	0.767	Kloss-Brandsatter et al. (2010)	Sanger sequencing	Complete
PCA050	Prostate carcinoma	G14207A	T156I	*MT-ND6*	0.831	Kloss-Brandsatter et al. (2010)	Sanger sequencing	Complete
PCA025	Prostate carcinoma	T14463C	T71A	*MT-ND6*	0.624	Kloss-Brandsatter et al. (2010)	Sanger sequencing	Complete
PCA019	Prostate carcinoma	G15243A	G166E	*MT-CYB*	0.933	Kloss-Brandsatter et al. (2010)	Sanger sequencing	Complete

3	Thyroid tumors	A5298G	I277V	*MT-ND2*	0.42	Abu-Amero et al. (2005)	Sanger sequencing	Complete
4	Thyroid tumors	5408delA	Frameshift	*MT-ND2*	NA	Abu-Amero et al. (2005)	Sanger sequencing	Complete
8	Thyroid tumors	G9948A	V248I	*MT-CO3*	0.627	Abu-Amero et al. (2005)	Sanger sequencing	Complete
2	Thyroid tumors	G11126A	E123K	*MT-ND4*	0.735	Abu-Amero et al. (2005)	Sanger sequencing	Complete
1	Thyroid tumors	A13514G	D393G	*MT-ND5*	0.818	Abu-Amero et al. (2005)	Sanger sequencing	Complete
9	Thyroid tumors	A14417G	V86A	*MT-ND6*	0.423	Abu-Amero et al. (2005)	Sanger sequencing	Complete
1	invasive mamma carcinoma upon mastectomy or tumorectomy	T12875C	I180T	*MT-ND5*	0.635	Fendt et al. (2011)	Sanger sequencing	Complete
7	invasive mamma carcinoma upon mastectomy or tumorectomy	T15341C	F199L	*MT-CYB*	0.717	Fendt et al. (2011)	Sanger sequencing	Complete
11	invasive mamma carcinoma upon mastectomy or tumorectomy	T12131C	S458P	*MT-ND4*	0.224	Fendt et al. (2011)	Sanger sequencing	Complete
12	invasive mamma carcinoma upon mastectomy or tumorectomy	G12803A	S156N	*MT-ND5*	0.65	Fendt et al. (2011)	Sanger sequencing	Complete
2	adult-onset leukaemia (acute lymphatic)	A15296G	I184V	*MT-CYB*	0.541	He et al. (2003)	Sanger sequencing	Complete
4	adult-onset leukaemia (acute lymphatic)	T11046C	L96P	*MT-ND4*	0.798	He et al. (2003)	Sanger sequencing	Complete
14	adult-onset leukaemia (chronic lympathic)	T4145C	F280S	*MT-ND1*	0.81	He et al. (2003)	Sanger sequencing	Complete
24	adult-onset leukaemia (chronic myeloid)	T15618C	V291A	*MT-CYB*	0.705	He et al. (2003)	Sanger sequencing	Complete
907	gastric cancer	G4996A	R176H	*MT-ND2*	0.909	Hung et al. (2010)	Sanger sequencing	Complete
917	gastric cancer	12418insA	Frameshift	*MT-ND5*	NA	Hung et al. (2010)	Sanger sequencing	Complete

1132	gastric cancer	G3697A	G131S	*MT-ND1*	0.854	Hung et al. (2010)	Sanger sequencing	Complete
37	lung cancer	T5215C	L249P	*MT-ND2*	0.712	Jin et al. (2007)	Sanger sequencing	Complete
6	lung cancer	A5466G	T333A	*MT-ND2*	0.533	Jin et al. (2007)	Sanger sequencing	Complete
29	lung cancer	A5914C	D4A	*MT-CO1*	0.248	Jin et al. (2007)	Sanger sequencing	Complete
23	lung cancer	G6075A	V58I	*MT-CO1*	0.597	Jin et al. (2007)	Sanger sequencing	Complete
47	lung cancer	A8108G	I175V	*MT-CO2*	0.562	Jin et al. (2007)	Sanger sequencing	Complete
18	lung cancer	T8843C	I106T	*MT-ATP6*	0.642	Jin et al. (2007)	Sanger sequencing	Complete
25	lung cancer	G9053A	S176N	*MT-ATP6*	0.37	Jin et al. (2007)	Sanger sequencing	Complete
15	lung cancer	G9525A	A107T	*MT-CO3*	0.594	Jin et al. (2007)	Sanger sequencing	Complete
61	lung cancer	T9877C	M224T	*MT-CO3*	0.408	Jin et al. (2007)	Sanger sequencing	Complete
36	lung cancer	G9984A	STOP	*MT-CO3*	NA	Jin et al. (2007)	Sanger sequencing	Complete
26	lung cancer	T10084C	I9T	*MT-ND3*	0.414	Jin et al. (2007)	Sanger sequencing	Complete
25	lung cancer	T11841C	L361P	*MT-ND4*	0.693	Jin et al. (2007)	Sanger sequencing	Complete
19	lung cancer	C12135T	S459F	*MT-ND4*	0.319	Jin et al. (2007)	Sanger sequencing	Complete
46	lung cancer	A12358G	T8A	*MT-ND5*	0.265	Jin et al. (2007)	Sanger sequencing	Complete
54	lung cancer	G13726A	A464T	*MT-ND5*	0.695	Jin et al. (2007)	Sanger sequencing	Complete
39	lung cancer	T13781C	I482T	*MT-ND5*	0.737	Jin et al. (2007)	Sanger sequencing	Complete
11	lung cancer	G14081A	G582D	*MT-ND5*	0.828	Jin et al. (2007)	Sanger sequencing	Complete
56	lung cancer	G15863A	E373K	*MT-CYB*	0.646	Jin et al. (2007)	Sanger sequencing	Complete
2	pilocity astrocytomas	A8704G	M60V	*MT-ATP6*	0.442	Lueth et al. (2009)	Sanger sequencing	Complete
5	pilocity astrocytomas	C15452A	L236I	*MT-CYB*	0.098	Lueth et al. (2009)	Sanger sequencing	Complete
16	pilocity astrocytomas	C6237A	L112M	*MT-CO1*	0.641	Lueth et al. (2009)	Sanger sequencing	Complete
2	conventional renal carcinoma	G4584A	A39T	*MT-ND2*	0.681	Meierhofer et al. (2006)	DHPLC followed by sanger sequencing	Complete
3	conventional renal carcinoma	A7423G	E507G	*MT-CO1*	0.524	Meierhofer et al. (2006)	DHPLC followed by sanger sequencing	Complete
4	conventional renal carcinoma	C12510A	D58E	*MT-ND5*	0.68	Meierhofer et al. (2006)	DHPLC followed by sanger sequencing	Complete
6	papillary renal carcinoma	T10579C	M37T	*MT-ND4L*	0.57	Meierhofer et al. (2006)	DHPLC followed by sanger	Complete

							sequencing	
B24	breast ductal carcinomas	G13708A	A458T	*MT-ND5*	0.409	Parrella et al. (2001)	Sanger sequencing	Complete
B32	breast ductal carcinomas	T12344A	M3K	*MT-ND5*	0.397	Parrella et al. (2001)	Sanger sequencing	Complete
B35	breast ductal carcinomas	G11900A	V381M	*MT-ND4*	0.675	Parrella et al. (2001)	Sanger sequencing	Complete
18	Prostate cancer	G5949A	G16X	*MT-CO1*	NA	Petros et al. (2005)	Sanger sequencing	COI only
	Prostate cancer	G6924T	A341S	*MT-CO1*	0.698	Petros et al. (2005)	Sanger sequencing	COI only
V478	colorectal tumours	T3308C	M1T	*MT-ND1*	0.577	Polyak et al. (1998)	Sanger sequencing	Complete
V429	colorectal tumours	G8009A	V142M	*MT-CO2*	0.581	Polyak et al. (1998)	Sanger sequencing	Complete
V429	colorectal tumours	G14985A	R80H	*MT-CYB*	0.833	Polyak et al. (1998)	Sanger sequencing	Complete
V429	colorectal tumours	T15572C	F276L	*MT-CYB*	0.69	Polyak et al. (1998)	Sanger sequencing	Complete
V441	colorectal tumours	G9948A	V248I	*MT-CO3*	0.627	Polyak et al. (1998)	Sanger sequencing	Complete
V456	colorectal tumours	T10563C	C32R	*MT-ND4L*	0.766	Polyak et al. (1998)	Sanger sequencing	Complete
V425	colorectal tumours	G6264A	G121X	*MT-CO1*	NA	Polyak et al. (1998)	Sanger sequencing	Complete
V425	colorectal tumours	12418insA	Frameshift	*MT-ND5*	NA	Polyak et al. (1998)	Sanger sequencing	Complete
112	breast cancer	T9131C	L202P	*MT-ATP6*	0.787	Tan et al. (2002)	Sanger sequencing	Complete
5	oral cancer	9485del	Frameshift	*MT-CO3*	NA	Tan et al. (2003)/Tan et al. (2004)	Sanger sequencing	Complete
18	oral cancer	A4986C	T173P	*MT-ND2*	0.774	Tan et al. (2003)/Tan et al. (2004)	Sanger sequencing	Complete
19	oral cancer	A5026G	H186R	*MT-ND2*	0.811	Tan et al. (2003)/Tan et al. (2004)	Sanger sequencing	Complete
E12	esophageal cancer	G10500A	A11T	*MT-ND4L*	0.64	Tan et al. (2006)	Sanger sequencing	Complete
E18	esophageal cancer	10941del TAACAACCCCC	110X	*MT-ND4*	NA	Tan et al. (2006)	Sanger sequencing	Complete
	primary fibrolamellar carcinomas	A4824G	T119A	*MT-ND2*	0.368	Vivekanandan et al. (2010)	Sanger sequencing	Complete
14	Hepatocellular carcinoma	3894-3960del66bp	Frameshift	*MT-ND1*	NA	Yin et al. (2010)	Sanger sequencing	Complete
20	Hepatocellular carcinoma	G9267A	A21T	*MT-CO3*	0.752	Yin et al. (2010)	Sanger sequencing	Complete
24	Hepatocellular carcinoma	T6787C	V295A	*MT-CO1*	0.69	Yin et al. (2010)	Sanger sequencing	Complete
24	Hepatocellular carcinoma	11032delA	Frameshift	*MT-ND4*	NA	Yin et al. (2010)	Sanger sequencing	Complete
81	Hepatocellular carcinoma	A11708G	I317V	*MT-ND4*	0.452	Yin et al. (2010)	Sanger sequencing	Complete
83	Hepatocellular carcinoma	G7976A	G131S	*MT-CO2*	0.651	Yin et al. (2010)	Sanger sequencing	Complete
98	Hepatocellular carcinoma	G3842A	W179X	*MT-ND1*	NA	Yin et al. (2010)	Sanger sequencing	Complete
99	Hepatocellular carcinoma	12418insA	Frameshift	*MT-ND5*	NA	Yin et al. (2010)	Sanger sequencing	Complete

1604	chromophobe renal cell carcinomas	13883delC	Frameshift	*MT-ND5*	NA	Nagy et al. (2002)	Sanger sequencing	Complete
1608	chromophobe renal cell carcinomas	10125insT	Frameshift	*MT-ND3*	NA	Nagy et al. (2002)	Sanger sequencing	Complete
844	Breast cancer	G4665A	A66T	*MT-ND2*	0.713	Zhu et al. (2005)	Sanger sequencing	Complete
697	Breast cancer	A8498G	K45E	*MT-ATP8*	0.409	Zhu et al. (2005)	Sanger sequencing	Complete
983	Breast cancer	T9885A	F227I	*MT-CO3*	0.826	Zhu et al. (2005)	Sanger sequencing	Complete
1026	Breast cancer	T9885A	F227I	*MT-CO3*	0.826	Zhu et al. (2005)	Sanger sequencing	Complete
911	Breast cancer	A11768G	T337A	*MT-ND4*	0.567	Zhu et al. (2005)	Sanger sequencing	Complete
988	Breast cancer	T13674G	N446K	*MT-ND5*	0.722	Zhu et al. (2005)	Sanger sequencing	Complete
738	Breast cancer	A15824G	T360A	*MT-CYB*	0.249	Zhu et al. (2005)	Sanger sequencing	Complete
884	Bladder cancer	T10321C	V88A	*MT-ND3*	0.409	Fliss et al. (2000)	Sanger sequencing	Complete
1678	Head and Neck Cancer	G11150A	A131T	*MT-ND4*	0.65	Fliss et al. (2000)	Sanger sequencing	Complete
126	Medulloblastoma	T11046C	L96P	*MT-ND4*	0.798	Wong et al. (2003)	Sanger	Complete
	Papillary thyroid carcinoma	A10398G	T114A	*MT-ND3*	0.17	Yeh et al. (2000)	Sanger	Complete
	Papillary thyroid carcinoma	G15179A	V145M	*MT-CYB*	0.715	Yeh et al. (2000)	Sanger	Complete
GC2	gastric cancer	G8572A	G16S	*MT-ATP6*	0.355	Bi et al. (2011)	Sanger	Complete
GC2	gastric cancer	G15777A	S344N	*MT-CYB*	0.522	Bi et al. (2011)	Sanger	Complete
GC3	gastric cancer	T15597C	V284A	*MT-CYB*	0.582	Bi et al. (2011)	Sanger	Complete
GC6	gastric cancer	G4632A	A55T	*MT-ND2*	0.82	Bi et al. (2011)	Sanger	Complete
BC155	Breast cancer	T14050C	S572P	*MT-ND5*	0.494	Shen et al. (2011)	Sanger	ND5 only
OV34	Ovarian carcinoma	G15761A	G339X	*MT-CYB*	NA	Liu et al. (2001)	Sanger	Complete
